# Sex disparities in tuberculosis outcomes: evidence from a multicenter Italian cohort (Italian South TB Network (ISTB-Net)

**DOI:** 10.1007/s15010-026-02725-x

**Published:** 2026-01-11

**Authors:** Francesco Di Gennaro, Alessandro Cornelli, Giacomo Guido, Rosa Buonamassa, Francesco Cavallin, Mariantonietta Pisaturo, Lorenzo Onorato, Federica Zimmerhofer, Giuseppe Bruno, Massimo Fasano, Agostina Pontarelli, Tiziana Iacovazzi, Luisa Frallonardo, Gianfranco Panico, Raffaella Libertone, Caterina Monari, Alessia Musto, Francesca Serapide, Mariangela Niglio, Sergio Cotugno, Roberta Papagni, Alberto Enrico Maraolo, Loredana Alessio, Giulio Viceconte, Giuseppina De Iaco, Aurelia Ricciardi, Rossana Lattanzio, Federica De Gregorio, Helen Linda Morrone, Ylenia Farinaccio, Gaetano Brindicci, Marinella Cibelli, Carmen Pellegrino, Giorgia Manco Cesari, Vito Spada, Paolo Tundo, Paola Mencarini, Carmen Rita Santoro, Giuliana Metrangolo, Annamaria Maci, Grazia Pietramatera, Gina Gualano, Salvatore Minniti, Giovanni Battista Buccoliero, Sergio Lo Caputo, Alessandra Prozzo, Sergio Carbonara, Antonio Cascio, Alessandro Russo, Ivan Gentile, Roberto Parrella, Fabrizio Palmieri, Nicola Coppola, Annalisa Saracino, Italian South T. B. Network (ISTB-Net)

**Affiliations:** 1 Department of Precision and Regenerative Medicine and Jonian Area (DiMePreJ), Clinic of Infectious Diseases, University of Bari Aldo Moro, Bari, BA Italy; 2 Department of Mental Health and Public Medicine, Section of Infectious Diseases, University of Campania Luigi Vanvitelli, Naples, Italy; 3 Department of Internal Medicine and Medical Therapy, University of Pavia (UniPV), Pavia, Italy; 4 Independent Statistician, Solagna, Italy; 5 Department of Health Promotion, Mother and Child Care, Internal Medicine and Medical Specialties “G D’Alessandro”, Infectious and Tropical Disease Unit, University of Palermo, Palermo, Italy; 6 Infectious Disease Department, Saint Giuseppe Moscati Hospital, Taranto, TA Italy; 7 UOC Malattie Infettive, ASL BA, PO Della Murgia “Fabio Perinei”, Altamura, BA Italy; 8 Respiratory Infectious Disease Unit, AORN Dei Colli, Cotugno Hospital, Naples, NA Italy; 9 Respiratory Infectious Diseases Unit, National Institute for Infectious Diseases Lazzaro Spallanzani-IRCCS, Rome, RM Italy; 10 UOC Malattie Infettive, Ospedale Vittorio Emanuele II, ASL BT, Bisceglie, BT Italy; 11 UOC Malattie Infettive, Ospedale Antonio Perrino, Brindisi, BR Italy; 12 Department of Medical and Surgical Sciences, Infectious and Tropical Disease Unit, ‘Magna Graecia’, University of Catanzaro, Catanzaro, Italy; 13 Department of Medical and Surgical Sciences, Infectious Diseases Unit, University of Foggia, Foggia, FG Italy; 14 USD Malattie Infettive E Tropicali, ASM Matera, Presidio Ospedaliero “Madonna Delle Grazie”, Matera, Italy; 15 Section of Infectious Diseases, Department of Clinical Medicine and Surgery, University of Naples “Federico II,”, Naples, Italy; 16 UOSVD Malattie Infettive, P.O. “A. Cardarelli”, Campobasso, CB Italy; 17 UOC Malattie Infettive, Santa Caterina Novella Hospital, Galatina, Italy; 18 UOC Malattie Infettive, Vito Fazzi Hospital, Lecce, Italy

**Keywords:** Tuberculosis, Sex, Migration, Health equity, Italy, Treatment outcomes, Hospital stay, Adherence

## Abstract

**Background:**

Sex disparities in tuberculosis (TB) outcomes are not well characterized, especially in high-income countries where social vulnerability and migration influence access to care. Although men globally experience a higher TB burden, the interaction between sex, migration, and social determinants is complex and extends beyond biological factors. This study evaluated sex differences in clinical and programmatic TB outcomes in a high-income European country with a significant substantial migrant population.

**Methods:**

A retrospective multicentre cohort study was conducted across 16 Infectious Diseases Units in seven Italian regions from (January 2021 to September 2025). Outcomes included time to sputum conversion (in pulmonary TB), length of hospital stay (LOS), adverse events (AEs) and their severity, incomplete treatment (defined as failure, death, or loss to follow-up), and loss to follow-up (LTFU). Mixed-effects models were applied using two prespecified adjustment sets: sex, centre, and core confounders (Model A); and sex, centre, and clinically relevant baseline imbalances (Model B). Sub-analyses examined the impact of migration status.

**Results:**

Of 982 TB patients, 229 (23.3%) were women and 753 (76.7%) were men. Women exhibited lower rates of smoking (24.4% vs 36.7%), diabetes (7.9% vs 15.8%), and COPD/bronchiectasis (4.5% vs 10.3%). The median sputum conversion time was 21 days for both sexes. Adjusted analysesindicated shorter LOS among women (Model A: − 22% [95%CI − 32 to − 10]; Model B: − 19% [95%CI − 28 to − 9]). Time to sputum conversion was slightly shorter in women in Model A (− 13%; 95%CI −23% to −1%) but not in Model B (− 9%; 95%CI −17% to 1%). The risk and severity of AEs were similar between sexes. In Model B, women had lower odds of incomplete treatment (OR 0.64 [95%CI 0.41 to 0.99]) and LTFU (OR 0.62 [95%CI 0.38 to 0.99]). Migrants experienced worse overall outcomes, but the effect of sex did not differ by migration status.

**Conclusion:**

Women had consistently shorter hospital stays and greater treatment continuity without increased toxicity, indicating that sex differences in TB outcomes are likely attributable to social and behavioural factors rather than biological differences. Supportive associative networks and non-governmental organisations may help reduce sex disparities, underscoring the importance of sex- and migration-responsive TB care models in Europe.

**Supplementary Information:**

The online version contains supplementary material available at 10.1007/s15010-026-02725-x.

## Introduction

Tuberculosis (TB) continues to be a leading global infectious cause of morbidity and mortality, with a disproportionate impact on men, socioeconomically disadvantaged populations, and individuals living in precarious conditions (1, 2). Worldwide, men represent nearly two-thirds of TB cases and deaths. This disparity is frequently attributed to gendered patterns of exposure, healthcare access, and health-seeking behaviour, rather than biological sex alone (1).

Recent analyses from Eastern Europe indicate that although men are more frequently affected by TB experience worse outcomes, much of this disparity is reduced after adjusting for social and behavioural factors (2).

In high-income countries such as Italy, where TB incidence is low but concentrated among socially vulnerable and migrant populations, understanding the influence of sex on treatment outcomes is essential for designing equitable and person-centred TB services (3, 4). Migrants, in particular, face multiple vulnerabilities, including linguistic, legal, and social challenges, which may intersect with sex to affect diagnostic delay, treatment adherence, and retention in care (4). However, multicentre evidence from Western and Southern Europe is limited, and the interaction between sex and migration has rarely been examined systematically.

A large multicentre cohort study was conducted across 16 Infectious Diseases Units in seven Italian regions to examine sex differences in clinical and programmatic TB outcomes, including sputum conversion, length of hospital stay (LOS), adverse events, treatment completion, and loss to follow-up (LTFU). The study also exploredhow these patterns differ between migrant and non-migrant patients.

The aim was to determine whether sex disparities persist within an equitable care context by situating the analysis in a universal healthcare system and a population characterised by significant migration.

## Materials and methods

### Study design

This retrospective, multicenter observational study was conducted in the Infectious and Tropical Disease Units of 16 referral hospitals across seven Italian regions: Puglia, Campania, Molise, Basilicata, Calabria, Sicily, and Lazio. Details regarding the study setting are provided in the Supplementary Material. The objective was to evaluate the impact of sex differences on tuberculosis-related outcomes.

### Patients

All patients diagnosed with TB at the participating centers between January 2021 and September 2025 were retrospectively included in the analysis.

### Outcome measures

The outcome measures included: (1) time to sputum conversion in pulmonary tuberculosis, (2) duration of hospital stay, (3) incidence and severity of adverse events related to anti-tuberculosis therapy, (4) incomplete treatments, encompassing treatment failure, mortality, and loss to follow-up, and (5) loss to follow-up. Tuberculosis outcomes were assessed according to the World Health Organization classification (5).

### Data collection

All data were retrospectively obtained from hospital charts and follow-up documentation, then compiled into an anonymized database for analysis. Collected data encompassed demographics, clinical characteristics, medical history, treatment details, adverse events, length of hospital stay, and follow-up information.

### Statistical analysis

Numerical variables were summarized as median and interquartile range (IQR), while categorical variables were presented as absolute and relative frequencies (percentages). Unadjusted analyses compared baseline characteristics and outcome measures between women and men using the Mann–Whitney test for numerical variables and either Fisher’s exact test or the Chi-Square test for categorical variables. Adjusted analyses assessed the association between sex and each outcome measure using mixed regression models that included sex, center, and either key confounders identified from prior knowledge (model A) or unbalanced clinically relevant variables (model B). Details of these models are provided in Supplementary Table 1. An exploratory sub-analysis examined whether the effect of sex on outcome measures differed between migrant and non-migrant patients, utilizing mixed regression models that incorporated sex, migrant status, their interaction, and center. All statistical tests were two-sided, and p-values less than 0.05 were considered statistically significant. Statistical analyses were performed using R version 4.5 (R Foundation for Statistical Computing, Vienna, Austria) (6).

### Ethics statement

Ethical approval was obtained from the local Ethics Committees (approval number 7401, 23/03/2024). Given the study's retrospective nature and the exclusive use of anonymized data, the requirement for written informed consent was waived.

## Results

### Demographics and clinical characteristics

A total of 982 tuberculosis (TB) patients were diagnosed at the participating centers between January 2021 and September 2025. The cohort comprised 229 women (23.3%) and 753 men (76.7%), who exhibited differences in certain demographic and clinical characteristics (Table 1). At diagnosis, women demonstrated lower rates of smoking (24.44% vs. 36.7%, *p* < 0.001), diabetes (7.9% vs. 15.8%, *p* = 0.003), and COPD/bronchiectasis (4.5% vs. 10.3%, *p* = 0.01) compared to men, whereas cough was more frequently reported among women (38.4% vs. 30.7%, *p* = 0.03). Drug resistance patterns also differed between women and men (Table 1).

**Table 1:** Demographics and clinical characteristics of TB patients stratified by sex

Variable	Women (*n* = 229)	Men (*n* = 753)	*p*-value
Age, years	42 (29–53)	39 (27–55)	0.39
Age 65 years or older	31/229 (13.5%)	109/753 (14.5%)	0.80
Migrants	178/229 (77.7%)	585/753 (77.7%)	0.99
WHO region:			0.47
AFR	74/178 (41.6%)	248/585 (42.4%)	
AMR	5/178 (2.8%)	7/585 (1.2%)	
EMR	22/178 (12.3%)	88/585 (15.0%)	
EUR	40/178 (22.5%)	112/585 (19.1%)	
SEAR	32/178 (18.0%)	118/585 (20.2%)	
WPR	5/178 (2.8%)	12/585 (2.1%)	
BMI:			0.90
Underweight	71/174 (40.8%)	228/535 (42.6%)	
Normal weight	93/174 (53.5%)	279/535 (52.2%)	
Overweight/obese	10/174 (5.7%)	28/535 (5.2%)	
Worker	134/227 (59.0%)	391/733 (53.3%)	0.15
Smoking habits	56/229 (24.4%)	276/753 (36.7%)	< 0.001
Diabetes	18/229 (7.9%)	119/753 (15.8%)	0.003
Hypertension	31/229 (13.5%)	107/753 (14.2%)	0.88
COPD/bronchiectasis	10/220 (4.5%)	71/692 (10.3%)	0.01
Chronic renal disease	10/229 (4.4%)	36/753 (4.8%)	0.93
HBV/HCV infection history	5/229 (2.2%)	41/753 (5.4%)	0.06
PLHIV	11/229 (4.8%)	21/753 (2.8%)	0.20
Cancer	3/229 (1.3%)	22/753 (2.9%)	0.26
Previous TB	7/229 (3.1%)	33/753 (4.4%)	0.48
Cough	88 /229 (38.4%)	231/753 (30.7%)	0.03
Chronic cough (at least 8 weeks)	38/229 (16.6%)	102/753 (13.5%)	0.29
Fever	48/229 (21.0%)	139/753 (18.5%)	0.45
Weight loss	42/229 (18.3%)	114/753 (15.1%)	0.29
Night sweats	24/229 (10.5%)	68/753 (9.0%)	0.60
Chest pain	27/229 (11.8%)	108/753 (14.3%)	0.38
Dyspnea	14/229 (6.1%)	65/753 (8.6%)	0.28
Hemoptysis	7/229 (3.1%)	51/753 (6.8%)	0.05
Patient diagnostic delay, days^a^	60 (15–200)	60 (15–175)	0.41
Health system diagnostic delay, days^b^	8 (3–30)	8 (3–32)	0.67
Diagnosis:			0.72
Confirmed TB	150/229 (74.2%)	570/753 (75.7%)	
Presumptive TB	59/229 (25.8%)	183/753 (24.3%)	
Pulmonary TB	183/229 (79.9%)	616/753 (81.8%)	0.71
Extrapulmonary TB	41/229 (17.9%)	124/753 (16.5%)	
Both	5/229 (2.2%)	13/753 (1.7%)	
Asymptomatic TB	110/229 (48.0%)	399/753 (53.0%)	0.22
TIMIKA score ^c^	65 (40–80)	60 (40–80)	0.76
Drug resistance:			0.04
Pan-susceptible	183/229 (79.5%)	578/753 (76.7%)	
Mono-resistant	34/229 (14.9%)	151/753 (20.1%)	
MDR	12/229 (5.2%)	17/753 (2.3%)	
preXDR/XDR	1/229 (0.4%)	7/753 (0.9%)	
Respiratory failure	52 (22.7%)	198 (26.3%)	0.31

Data summarized as n (%) or median (IQR). Data not available in ^a^4, ^b^5 and ^c^209 patients. WHO region included African Region (AFR), Region of the Americas (AMR), Eastern Mediterranean Region (EMR), European Region (EUR), South-Eastern Asian Region (SEAR), Western Pacific Region (WPR)

### Time of sputum conversion

**Fig. 1:**
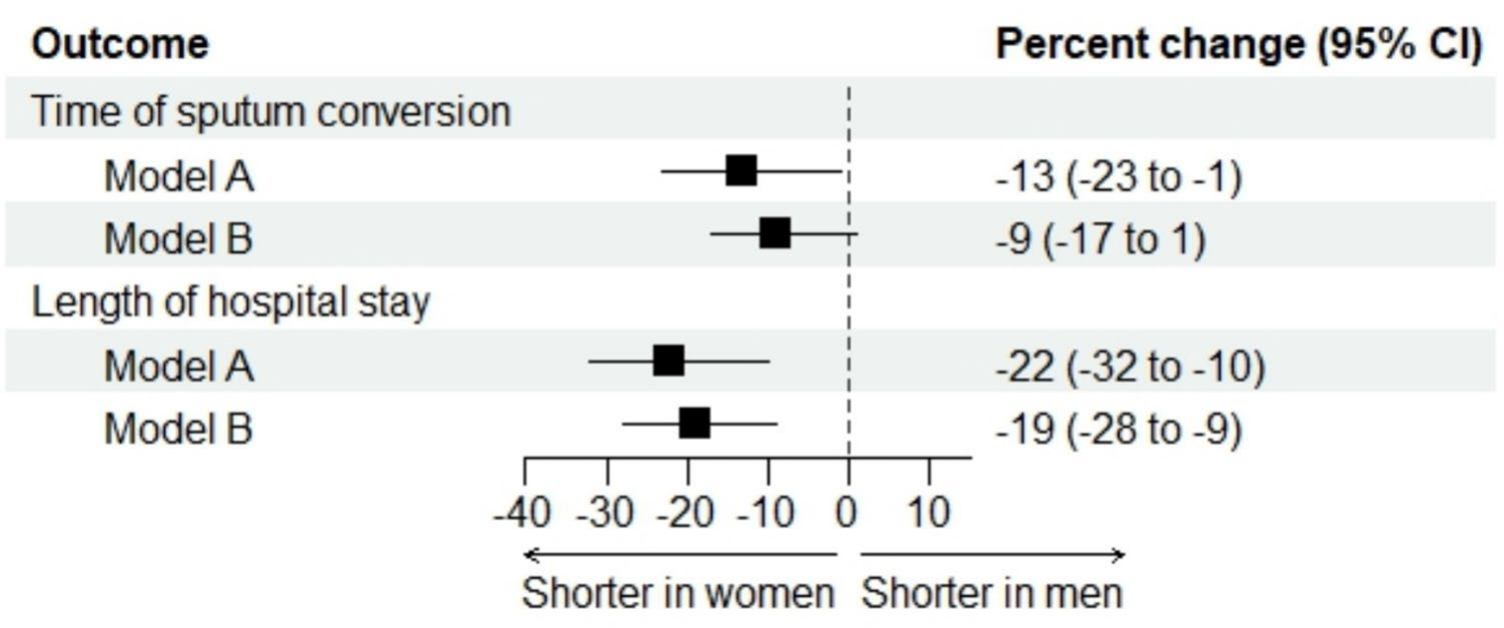
Time of sputum conversion (days) and length of hospital stay (days) in women vs. men: forest plot of the adjusted analyses. The model A included sex, center and key confounders based on prior knowledge; the model B included sex, center and unbalanced characteristics at baseline

Among 817 patients with pulmonary tuberculosis, the median time to sputum conversion was 21 days for both sexes (Table 2). After adjustment for key confounders (model A), women exhibited a 13% shorter time to sputum conversion compared to men (95% confidence interval 1% to 23%; *p* = 0.03) (Fig. 1). When adjusting for unbalanced confounders (model B), women demonstrated a 9% reduction in time to sputum conversion compared to men (95% confidence interval: -1% to 17%; *p* = 0.08) (Fig. 1).

**Table 2:** Outcome measures of TB patients stratified by sex

	Women (n = 229)	Men (*n* = 753)	*p*-value
Time of sputum conversion (in pulmonary TB), days ^a^	21 (14–29)	21 (14–30)	0.36
Length of hospital stay, days ^b^	28 (18–55)	35 (21–70)	< 0.001
Adverse events	50/229 (21.8%)	221/753 (29.3%)	0.03
Severity of the adverse events:			0.36
Mild	22/50 (44.0%)	74/221 (33.5%)	
Moderate	18/50 (36.0%)	90/221 (40.7%)	
Severe	10/50 (20.0%)	57/221 (25.8%)	
Incomplete treatment	33/229 (14.4%)	194/753 (25.7%)	< 0.001
Loss to follow-up	29/229 (12.7%)	175/753 (23.2%)	< 0.001

Data summarized as n (%) or median (IQR). Data were not available in ^a^233 and ^b^33 patients. Adverse events included: hepatitis (*n* = 144), gastrointestinal (*n* = 15), cutaneous (*n* = 30), neuropathy (*n* = 4), visual impairment (*n* = 13), hearing impairment (*n* = 6), renal failure (*n* = 23), more than one symptom (*n* = 16), QT prolongation (*n* = 9), generalized malaise (*n* = 11)

### Length of hospital stay

The median length of hospital stay was 28 days for women and 35 days for men (Table 2). After adjustment for key confounders (model A), the length of hospital stay was, on average, 22% shorter in women compared to men (95% confidence interval 10% to 32%; *p* < 0.001) (Fig. 1). When adjusting for unbalanced confounders (model B), the length of hospital stay was, on average, 19% shorter in women compared to men (95% confidence interval 9% to 28%; *p* < 0.001) (Fig. 1).

### Occurrence of adverse events

**Fig. 2:**
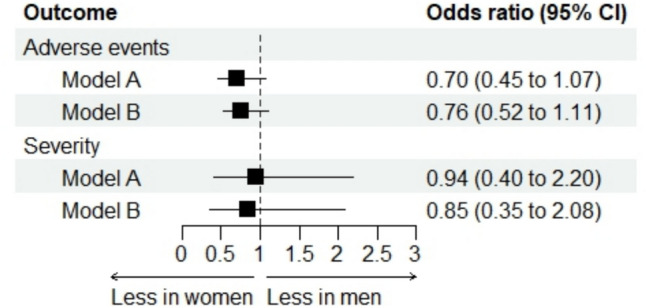
Occurrence and severity of adverse events in women vs. men: forest plot of the adjusted analyses. The model A included sex, center and key confounders based on prior knowledge; the model B included sex, center and unbalanced characteristics at baseline

Adverse events are summarized in Supplementary Table 2. The incidence of adverse events was 21.8% in women and 29.3% in men (Table 2). After adjustment for key confounders (model A), there was no statistically significant difference in adverse event occurrence between women and men (odds ratio 0.70, 95% confidence interval 0.45 to 1.07; *p* = 0.10) (Fig. 2). Similarly, adjustment for unbalanced confounders (model B) did not reveal a statistically significant difference (odds ratio 0.76, 95% confidence interval 0.52 to 1.11; *p* = 0.15) (Fig. 2).

### Severity of adverse events

No significant differences in theseverity of adverse events was observed between women and men (Table 2). After adjustment for key confounders (model A), the odds of severe adverse events remained statistically similar between women and men (odds ratio 0.94, 95% confidence interval 0.40 to 2.20; *p* = 0.89) (Fig. 2). When adjusting for unbalanced confounders (model B), the results were consistent (odds ratio 0.85, 95% confidence interval 0.35 to 2.08; *p* = 0.73) (Fig. 2).

### Incomplete treatments

**Fig. 3:**
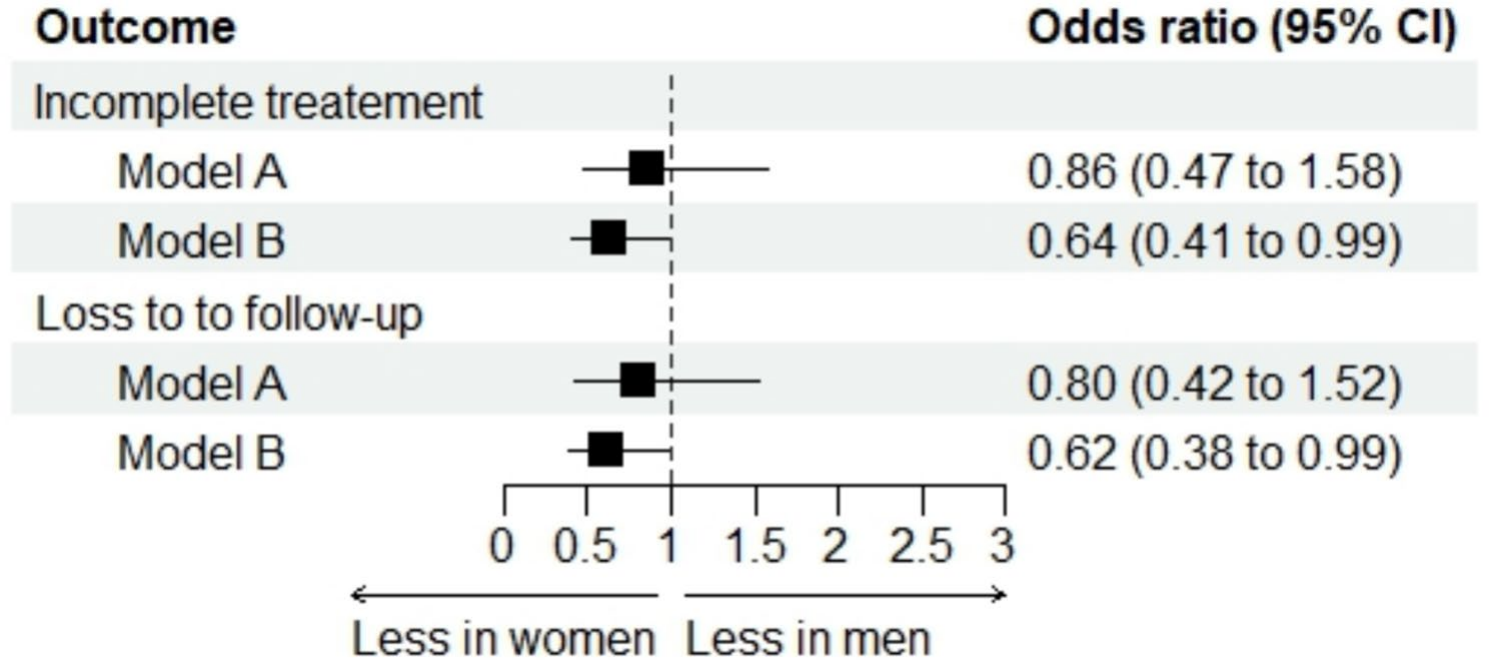
Incomplete treatments and loss to follow-up in women vs. men: forest plot of the adjusted analyses. The model A included sex, center and key confounders based on prior knowledge; the model B included sex, center and unbalanced characteristics at baseline

The proportion of incomplete treatments was 14.4% among women and 25.7% among men (Table 2). After adjustment for key confounders (model A), the difference in incomplete treatments between women and men was not statistically significant (odds ratio 0.86, 95% confidence interval 0.47–1.58; *p* = 0.63) (Fig. 3). When adjusting for unbalanced confounders (model B), incomplete treatments were significantly less likely in women than men (odds ratio 0.64, 95% confidence interval 0.41–0.99; *p* = 0.04) (Fig. 3).

### Loss to follow-up

Loss to follow-up occurred in 12.7% of women and 23.2% of men (Table 2). After adjustment for key confounders (model A), there was no statistically significant difference in loss to follow-up between women and men (odds ratio 0.80, 95% confidence interval 0.42–1.52; *p* = 0.49) (Fig. 3). When adjusting for unbalanced confounders (model B), women were less likely than men to be lost to follow-up (odds ratio 0.62, 95% confidence interval 0.38–0.99; *p* = 0.04) (Fig. 3).

### Impact of sex in migrants and non-migrant patients

Migrants experienced a 42% longer time of sputum conversion (95% confidence interval 26%–60%, *p* < 0.001) and a 45% longer hospital stay (95% confidence interval 20% to 70%, *p* < 0.001) than non-migrants patients. They also had a higher occurrence of adverse events (odds ratio 1.98, 95% confidence interval 1.27–3.09; *p* = 0.002) but lower proportion of severe adverse events (odds ratio 0.36, 95% confidence interval 0.15–0.87; *p* = 0.02). Additionally, migrants had more incomplete treatments (odds ratio 3.28, 95% confidence interval 1.97–5.47; *p* < 0.001) and were more frequently lost to follow-up (odds ratio 3.66, 95% confidence interval 2.12–6.30; *p* < 0.001). Migrant status did not statistically influence the impact of sex (Supplementary Material).

## Discussion

In this multicenter cohort study including almost one thousand TB patients, clear sex differences were observed between women and men at baseline. Specifically, women were less likely than men to report smoking, diabetes, and chronic respiratory diseases while, cough was more frequently reported among women at diagnosis. These results align with previous evidence behavioral and lifestyle risk factors are more common in men, which may contribute to their increased susceptibility to TB infection and poorer outcomes (7, 8). In contrast, the higher frequency of symptom reporting among women may reflect differences in care-seeking behaviour rather than actual disease severity.

Drug-resistance profiles varied by sex, with women exhibiting higher proportions of pan-susceptible and multidrug-resistant tuberculosis (MDR-TB), while men demonstrated a greater prevalence of mono-resistant forms. This disparity may be attributed to hormonal influences and comorbidity-related factors. In women, sex hormones modulate hepatic enzyme activity, thereby affecting the metabolism of isoniazid and rifampicin, higher plasma drug concentrations and a reduced risk of subtherapeutic levels (9). Recent research indicates that serum rifampicin levels in men are more frequently below the therapeutic range, even after dose adjustments (10). Additionally, women are typically less exposed to risk factors such as HIV, diabetes, chronic alcohol use, smoking, prolonged illness duration, and poor treatment adherence. These factors, more prevalent among men, increase the likelihood of treatment failure and drug resistance (11, 12).

A substantial proportion of individuals with resistant TB in this study were male migrants. These findings may be attributed to their increased exposure to high-risk environments for TB transmission, including prisons, shelters, and overcrowded workplaces, where multidrug-resistant strains are more prevalent (13). Additionally, within the context of migration, male migrants are more frequently subjected to detention or prolonged stays in transit camps compared to women, a pattern that reflects gendered migration trends and labor-related mobility (14).

The literature does not consistently reflect these findings. A 2020 meta-analysis reported no global or national association between sex and the risk of MDR/RR-TB in most countries. Exceptions included certain former Soviet states, where men exhibited a higher risk, and countries with TB burdens concentrated among foreign-born populations, where women had a higher risk (15). Given the limited and context-specific nature of studies stratifying drug resistance by sex, the present data offer a valuable foundation for further investigation of this issue.

After adjustment for known confounders, women had a shorter time to sputum conversion; however, this finding was not confirmed after controlling for baseline imbalances. Multiple studies have also reported more rapid sputum conversion in women compared to men (16, 17). This difference may be attributable to the higher prevalence of comorbidities and more severe disease presentations, such as cavitary lesions, in men, both of which can impede bacteriological clearance.

Within the studied cohort, women consistently experienced shorter hospital stays, fewer excess adverse events, and lower rates of loss to follow-up and incomplete treatment than men, even after adjustment for baseline imbalances. Microbiological responses were similar between sexes, indicating that the observed advantages in programmatic outcomes among women are more likely attributable to contextual, behavioural, and social determinants of care rather than biological differences.

Occupational mobility may contribute to the higher rate of incomplete treatment observed among men. In Southern Italy, for instance, migrant men frequently work as seasonal agricultural laborers, resides temporarily in resettlement camps and frequently relocating within or outside Italy to seek employment opportunities (18). Such mobility can reduce family support and disrupt the continuity of care, leading to treatment interruptions. These observations are consistent with WHO and ECDC data, which indicate higher treatment completion rates among women across Europe, including within migrant populations (19, 20).

Migration remains a key structural determinant of TB vulnerability in Europe. In this cohort, migrants experienced worse outcomes, including longer hospitalization, more adverse events, and higher rates of incomplete treatment and loss to follow-up, which confirms persistent inequities even within high-income healthcare systems. These results are consistent with other studies in Italy and across Europe, that report ongoing inequalities in healthcare access between migrant and non-migrant populations (21–23). However, the sex gradient did not differ significantly by migration status; male disadvantage persisted among both migrant and non-migrant groups. One plausible explanation is presence of social and associative networks that support migrant women in Italy and Southern Europe.

Community-based and faith-based organisations, NGOs, and voluntary networks frequently oftenprioritise women and children, by providing linguistic mediation, social assistance, and healthcare navigation from the point of arrival. This focus on sex prioritization, as documented by WHO and IOM reports (14), arises from the recognition that migrant women are disproportionately exposed to violence, exploitation, and psychological trauma during migration. Paradoxically, these vulnerabilities can lead to targeted outreach that improves healthcare access and treatment adherence once individuals are within the healthcare system.

These findings may, at least in part, reflect the protective effect of associative networks, which buffer certain structural vulnerabilities that could otherwise worsen outcomes. In contrast, migrant men who are often more socially isolated and less reached by such initiatives, may remain at greater risk of incomplete care and disengagement (24).

These results underscore the need for sex-responsive and migration-sensitive TB programmes that address modifiable social and behavioural determinants. First, interventions specifically targeting men, such as tailored adherence counselling, screening for alcohol and tobacco use, and also active digital follow-up, should be prioritised. Second, integrating existing community and NGO networks into formal TB care pathways may extend the benefits of social mediation observed among women to other vulnerable groups. Third, routine sex-disaggregated monitoring of key programmatic indicators, including treatment initiation, completion, and loss to follow-up, should become standard practice across European TB services. Finally, partnerships between public health systems and civil society are essential for achieving equitable TB outcomes in settings characterised by social complexity and migration.

This study has several limitations. The retrospective design precludes causal inference, and residual confounding may persist despite careful adjustment. Small subgroup sizes may have reduced the ability to detect certain effects, as indicated by wide confidence intervals. Likewise, we cannot exclude Additionally, potential sex–migration interactions cannot be excluded due to the relatively small number of non-migrant patients in the cohort. Finally, as this multicentre study was conducted in a high-income country, the generalisability of the findings to low- and middle-income settings with higher TB burden is limited.

In conclusion, our findings highlight that sex continues to shape tuberculosis outcomes even within universal healthcare systems. Women experienced shorter hospital stays and greater treatment continuity, whereas men had higher rates of loss to follow-up and incomplete care. These disparities appear to stem less from biological factors than from social and behavioural determinants, including migration-related vulnerabilities. Their persistence despite universal access underscores that achieving TB equity requires addressing sex and migration-linked social realities, not only biomedical barriers. Strengthening sex-responsive, community-based, and migrant-sensitive TB programmes is essential to achieving equitable outcomes across Europe.

In summary, the findings indicate that sex remains a significant determinant of tuberculosis outcomes, even within universal healthcare systems. Women had shorter hospital stays and more consistent treatment, while men exhibited higher rates of loss to follow-up and incomplete care. These disparities are primarily attributed to social and behavioural determinants, such as migration-related vulnerabilities, rather than to biological factors. The persistence of these inequities despite universal access demonstrates that achieving tuberculosis equity necessitates addressing sex- and migration-related social factors in addition to biomedical barriers. Enhancing sex-responsive, community-based, and migrant-sensitive tuberculosis programmes is crucial for promoting equitable outcomes across Europe.

## Supplementary Information

Below is the link to the electronic supplementary material.

Supplementary file1 (DOCX 23 KB)

## Data Availability

No datasets were generated or analysed during the current study.
