# Supplementary material for: Sex disparities in tuberculosis outcomes: evidence from a multicenter Italian cohort (Italian South TB Network (ISTB-Net)
Source: Infection. 2026 Jan 11;54(2):895–903. doi: 10.1007/s15010-026-02725-x (PMC13021756; doi:10.1007/s15010-026-02725-x)
Supplement: Supplementary file 1 — Supplementary file1 (DOCX 23 KB) [file 15010_2026_2725_MOESM1_ESM.docx]

SUPPLEMENTARY MATERIAL

**Participating Centers and Study Network**

This multicentre study was conducted across 16 Infectious Diseases Units located in seven Italian regions—Puglia, Campania, Sicilia, Basilicata, Molise, Lazio, and Calabria—representing the central and southern areas of Italy.
The network included seven university-affiliated (teaching) hospitals, eight regional or provincial (non-teaching) hospitals, and one national infectious diseases institute, thereby ensuring a balanced representation of referral and community-based TB care facilities.

These regions share broadly comparable socioeconomic and demographic profiles, characterised by similar healthcare structures and access pathways for TB patients. All centres serve populations with overlapping migration and labour-related mobility patterns, including a significant presence of agricultural migrant workers. This geographical and social homogeneity supports comparability of findings across sites while reflecting the vulnerabilities typical of TB-affected populations in southern Italy.

**Puglia**

Department of Precision and Regenerative Medicine and Jonian Area (DiMePreJ), Clinic of Infectious Diseases, University of Bari Aldo Moro, Bari (Teaching Hospital)

Infectious Disease Department, Saint Giuseppe Moscati Hospital, Taranto (Non-teaching Hospital)

UOC Malattie Infettive, ASL BA, PO Della Murgia “Fabio Perinei”, Altamura (Non-teaching Hospital)

UOC Malattie Infettive, Ospedale Vittorio Emanuele II, ASL BT, Bisceglie (Non-teaching Hospital)

UOC Malattie Infettive, Ospedale Antonio Perrino, Brindisi (Non-teaching Hospital)

Department of Medical and Surgical Sciences, Infectious Diseases Unit, University of Foggia, Foggia (Teaching Hospital)

UOC Malattie Infettive, Vito Fazzi Hospital, Lecce (Non-teaching Hospital)

UOC Malattie Infettive, Santa Caterina Novella Hospital, Galatina (Non-teaching Hospital)

**Campania**

Department of Mental Health and Public Medicine, Section of Infectious Diseases, University of Campania Luigi Vanvitelli, Naples (Teaching Hospital)

Respiratory Infectious Disease Unit, AORN dei Colli, Cotugno Hospital, Naples (Non-teaching Hospital)

Department of Clinical Medicine and Surgery, University of Naples Federico II, Naples (Teaching Hospital)

**Sicilia**

Department of Health Promotion, Mother and Child Care, Internal Medicine and Medical Specialties “G. D’Alessandro”, Infectious and Tropical Disease Unit, University of Palermo, Palermo (Teaching Hospital)

**Basilicata**

UOC Malattie Infettive, Ospedale Madonna delle Grazie, Matera (Non-teaching Hospital)

**Molise**

UOSVD Malattie Infettive, “A. Cardarelli” Hospital, Campobasso (Non-teaching Hospital)

**Lazio**

Respiratory Infectious Diseases Unit, National Institute for Infectious Diseases Lazzaro Spallanzani – IRCCS, Rome (National Institute / Teaching)

**Calabria**

Department of Medical and Surgical Sciences, Infectious and Tropical Disease Unit, 'Magna Graecia' University of Catanzaro, Catanzaro (Teaching Hospital)

All participating centres are located in regions with comparable socioeconomic indicators, healthcare infrastructures, and migration-related dynamics. The southern Italian context, characterised by agricultural labour migration and social vulnerability, provides a consistent background for interpreting TB outcomes across sites.

***Description of the regression models***

In the main analysis, the adjusted analysis investigated the association between sex and each outcome measure using mixed regression models with two set of covariates, according to the following rationale.

The model A accounted for the multicenter design and the possible effect of key confounders; hence, the independent variables of model A included sex, center and other key confounders based on prior knowledge (which were chosen according to each outcome measure).

The model B accounted for the multicenter design and the possible effect of unbalanced characteristics between females and males; hence, the independent variables of the model B included sex, center and clinically relevant variables that were unbalanced at baseline between females and males (smoking habits, diabetes, COPD/bronchiectasis, cough and drug resistance). Full details are summarized in Supplementary Table 1.

Supplementary Table 1. Description of the regression models in the adjusted analysis of the outcome measures

| Dependent variable | Name | Model | Main independent variable | Other independent variables | Reported effect size of the main independent variable |
| --- | --- | --- | --- | --- | --- |
| Time of sputum conversion (in pulmonary TB) | Model A | Linear mixed-effect model with logarithmic transformation | Sex: women vs. men (fixed effect) | -center (random effect),  - age, total diagnostic delay, TIMIKA score, comorbidity status (diabetes, hypertension, HIV), respiratory failure, adverse event, underweight (fixed effects) | Percent change (with 95% confidence interval) in time of sputum conversion on average |
|  | Model B | Linear mixed-effect model with logarithmic transformation | Sex: women vs. men (fixed effect) | -center (random effect),  -smoking habits, diabetes, COPD/bronchiectasis, cough and drug resistance (fixed effects) | Percent change (with 95% confidence interval) in time of sputum conversion on average |
| Length of hospital stay | Model A | Linear mixed-effect model with logarithmic transformation | Sex: women vs. men (fixed effect) | -center (random effect),  - age, TIMIKA score, respiratory failure, migrant status, drug resistance, adverse event (fixed effects) | Percent change (with 95% confidence interval) in length of hospital stay on average |
|  | Model B | Linear mixed-effect model with logarithmic transformation | Sex: women vs. men (fixed effect) | -center (random effect),  -smoking habits, diabetes, COPD/bronchiectasis, cough and drug resistance (fixed effects) | Percent change (with 95% confidence interval) in length of hospital stay on average |
| Occurrence of adverse events | Model A | Logistic mixed-effect model | Sex: women vs. men (fixed effect) | -center (random effect),  - age, migrant status, drug resistance, comorbidity status (diabetes, hypertension, HIV, chronic renal disease), underweight (fixed effects) | Odds ratios with 95% confidence interval |
|  | Model B | Logistic mixed-effect model | Sex: women vs. men (fixed effect) | -center (random effect),  -smoking habits, diabetes, COPD/bronchiectasis, cough and drug resistance (fixed effects) | Odds ratios with 95% confidence interval |
| Severity of adverse events | Model A | Logistic mixed-effect model | Sex: women vs. men (fixed effect) | -center (random effect),  - age, migrant status, drug resistance, comorbidity status (diabetes, hypertension, HIV, chronic renal disease) (fixed effects) | Odds ratios with 95% confidence interval |
|  | Model B | Logistic mixed-effect model | Sex: women vs. men (fixed effect) | -center (random effect),  -smoking habits, diabetes, COPD/bronchiectasis, cough and drug resistance (fixed effects) | Odds ratios with 95% confidence interval |
| Incomplete treatments | Model A | Logistic mixed-effect model | Sex: women vs. men (fixed effect) | -center (random effect),  -age, TIMIKA score, migrant status, total diagnostic delay, comorbidity status (diabetes, hypertension, HIV), respiratory failure, adverse event, underweight (fixed effects) | Odds ratios with 95% confidence interval |
|  | Model B | Logistic mixed-effect model | Sex: women vs. men (fixed effect) | -center (random effect),  -smoking habits, diabetes, COPD/bronchiectasis, cough and drug resistance (fixed effects) | Odds ratios with 95% confidence interval |
| Loss to follow-up | Model A | Logistic mixed-effect model | Sex: women vs. men (fixed effect) | -center (random effect),  -age, TIMIKA score, migrant status, total diagnostic delay, comorbidity status (diabetes, hypertension, HIV), respiratory failure, adverse event, underweight (fixed effects) | Odds ratios with 95% confidence interval |
|  | Model B | Logistic mixed-effect model | Sex: women vs. men (fixed effect) | -center (random effect),  -smoking habits, diabetes, COPD/bronchiectasis, cough and drug resistance (fixed effects) | Odds ratios with 95% confidence interval |

***Type and severity of adverse events***

Type and severity of adverse events in the study cohort are summarized in Supplementary Table 2.

Supplementary Table 2. Type and severity of adverse events

| Adverse events | Total | Mild | Moderate | Severe |
| --- | --- | --- | --- | --- |
| Hepatitis | 144 | 49 | 63 | 32 |
| Gastrointestinal | 15 | 8 | 6 | 1 |
| Cutaneous | 30 | 14 | 13 | 3 |
| Neuropathy | 4 | 0 | 2 | 2 |
| Visual impairment | 13 | 2 | 7 | 4 |
| Hearing impairment | 6 | 4 | 0 | 2 |
| Renal failure | 23 | 9 | 6 | 8 |
| More than one symptom | 16 | 3 | 4 | 9 |
| QT prolongation | 9 | 0 | 5 | 4 |
| Generalized malaise | 11 | 7 | 2 | 2 |

Data summarized as n of patients.

***Impact of sex in migrants and non-migrant patients***

Time of sputum conversion in pulmonary TB was 42% longer in migrants vs. non-migrant patients(95% confidence interval 26% to 60%, p<0.001), but being migrant did not statistically influence the impact of sex (p=0.62).

The length of hospital stay was 45% longer in migrants vs. non-migrant patients (95% confidence interval 27% to 70%, p<0.001), but being migrant did not statistically influence the impact of sex (p=0.67).

The occurrence of adverse events was higher in migrants vs. non-migrant patients (odds ratio 1.98, 95% confidence interval 1.27 to 3.09; p=0.002), but being migrant did not statistically influence the impact of sex (p=0.40).

The proportion of severe adverse events was lower in migrants vs. non-migrant patients (odds ratio 0.36, 95% confidence interval 0.15 to 0.87; p=0.02), but being migrant did not statistically influence the impact of sex (p=0.80).

Incomplete treatments were more common in migrants vs. non-migrant patients (odds ratio 3.28, 95% confidence interval 1.97 to 5.47; p<0.001), but being migrant did not statistically influence the impact of sex (p=0.46).

Loss to follow-up was more frequent in migrants vs. non-migrant patients (odds ratio 3.66, 95% confidence interval 2.12 to 6.30; p<0.001), but being migrant did not statistically influence the impact of sex (p=0.97).
